# PpGATA21 Enhances the Expression of *PpGA2ox7* to Regulate the Mechanism of *Cerasus humilis* Rootstock-Mediated Dwarf in Peach Trees

**DOI:** 10.3390/ijms25137402

**Published:** 2024-07-05

**Authors:** Xiuzhen Li, Ruxin Wang, Yuman Wang, Xueqiang Li, Qiaofang Shi, Yihe Yu

**Affiliations:** 1 College of Horticulture and Plant Protection, Henan University of Science and Technology, Luoyang 471023, China; 9902551@haust.edu.cn (X.L.); wangrx0126@163.com (R.W.); 230320191100@stu.haust.edu.cn (Y.W.); lixq@haust.edu.cn (X.L.); 9906384@haust.edu.cn (Q.S.); 2 Henan Provincial Engineering Research Center on Characteristic Berry Germplasm Innovation & Utilization, Luoyang 471023, China

**Keywords:** *Cerasus humilis*, dwarfing rootstock, gibberellin, PpGATA21, GA 2-oxidase

## Abstract

Dwarfing rootstocks enhance planting density, lower tree height, and reduce both labor in peach production. *Cerasus humilis* is distinguished by its dwarf stature, rapid growth, and robust fruiting capabilities, presenting substantial potential for further development. In this study, Ruipan 4 was used as the scion and grafted onto *Amygdalus persica* and *Cerasus humilis*, respectively. The results indicate that compared to grafting combination R/M (Ruipan 4/*Amygdalus persica*), grafting combination R/O (Ruipan 4/*Cerasus humilis*) plants show a significant reduction in height and a significant increase in flower buds. RNA-seq indicates that genes related to gibberellin (GA) and auxin metabolism are involved in the dwarfing process of scions mediated by *C. humilis*. The expression levels of the GA metabolism-related gene *PpGA2ox7* significantly increased in R/O and are strongly correlated with plant height, branch length, and internode length. Furthermore, GA levels were significantly reduced in R/O. The transcription factor PpGATA21 was identified through yeast one-hybrid screening of the *PpGA2ox7* promoter. Yeast one-hybrid (Y1H) and dual-luciferase reporter (DLR) demonstrate that PpGATA21 can bind to the promoter of *PpGA2ox7* and activate its expression. Overall, PpGATA21 activates the expression of the GA-related gene *PpGA2ox7*, resulting in reduced GA levels and consequent dwarfing of plants mediated by *C. humilis.* This study provides new insights into the mechanisms of *C. humilis* and offers a scientific foundation for the dwarfing and high-density cultivation of peach trees.

## 1. Introduction

Peach (*Prunus persica* L. Batsch) is a globally important economic fruit. However, peach trees exhibit vigorous growth, produce numerous branches, and grow rapidly in the early stages, leading to high management and labor costs. With the establishment of modern standardized orchards, growers need to enhance standardized cultivation techniques for peach trees and the mechanization of orchard management [1,2]. The utilization of dwarf rootstocks can facilitate the transition of fruit trees from vegetative growth to reproductive growth, leading to earlier flowering and fruiting, enhancing early economic returns, and improving fruit quality [3,4]. Furthermore, the compact size of dwarf rootstocks makes them ideal for high-density planting, simplifying management during production [5]. Therefore, to align with the development trends of modern orchards, selecting dwarfing rootstocks, controlling tree vigor, and adopting low-density cultivation techniques have become primary objectives in peach tree breeding. Grafting is widely employed in horticulture to enhance plant growth characteristics, including improved tolerance, fruit quality, and dwarfing [6,7]. Research has demonstrated that macromolecules transported via vascular tissue between rootstock and scion play a crucial role in graft-induced phenotypic changes [8]. *PbWoxT1* of tobacco rootstocks can be transferred to wild-type tobacco scions, influencing flower morphology [9]. Expression of *CcFT3* in the phloem tissues of citrus can induce precocious flowering in grafted scions [10]. However, the molecular mechanisms underlying the interactions between rootstocks and scions remain largely unknown.

The selection of peach rootstock significantly influences key traits of peach scion varieties and greatly affects the profitability of peach orchards [11,12,13]. Additionally, introducing dwarfing traits can help control the growth potential of cash crops, including fruit trees [14]. Currently, grafting seedlings is the predominant method used in peach tree production, with *Amygdalus davidiana* (*Carr.*) *Yu* serving as a common rootstock for peach trees [1]. Differences in carbon allocation among various rootstocks influence the reduction of stem elongation [15]. Reduced canopy development (stem elongation) alters the light environment by decreasing intra-canopy shade for developing fruits, which in turn enhances fruit quality [16]. Therefore, the optimal selection and use of dwarfing rootstocks for peaches can enhance light availability and the uniformity of light distribution within the canopy, thereby improving fruit quality and reducing both management and labor costs in orchards [17]. *Cerasus humilis*, also known as the Chinese dwarf cherry or ‘calcium fruit’, is a fruit-bearing shrub native to northern China [18]. It is characterized by its short stature, rapid growth, and strong fruiting ability. Research has demonstrated that *C. humilis* can upregulate the expression of growth-related genes such as *PpYucca5*, *PpYucca2*, and *PpYucca6*, thereby reducing auxin levels and achieving a dwarfing effect [1]. However, the molecular mechanisms of dwarfing induced by *C. humilis* rootstock is still poorly understood. 

Hormonal regulation is regarded as the mechanism through which rootstocks impact scion viability by modulating chemical signals from root tips [7]. Alterations in cell elongation and division, controlled by various plant hormones including auxins, cytokinins, gibberellins (GAs), abscisic acid, and brassinosteroids, result in a dwarf phenotype [15,19,20]. GAs are essential for plant vascular development, cambial function, and the growth of xylem and phloem tissues [21]. There is significant evidence that metabolism of GA is crucial in causing dwarfing of plant shoots [22,23]. GA 2-oxidases (GA2oxs) are key enzymes in GA metabolism that degrade the active gibberellins GA1 and GA4 into the inactive forms GA8 and GA34. This transformation is a critical process in the regulation of biologically active GA levels in plants [24]. Currently, only a select few gibberellin molecules such as GA1, GA3, GA4, and GA7 exhibit biological activity in plants.

In this study, the mechanism by which *C. humilis* mediates dwarfing in peach trees through the regulation of GA metabolism was analyzed. This study aims to explain the mechanism by which *C. humilis* rootstocks influence the dwarfing of peach trees, providing a robust theoretical basis for using these rootstocks to regulate tree height.

## 2. Result

### 2.1. C. humilis Significantly Reduces the Height of Scions

**Figure 1:**
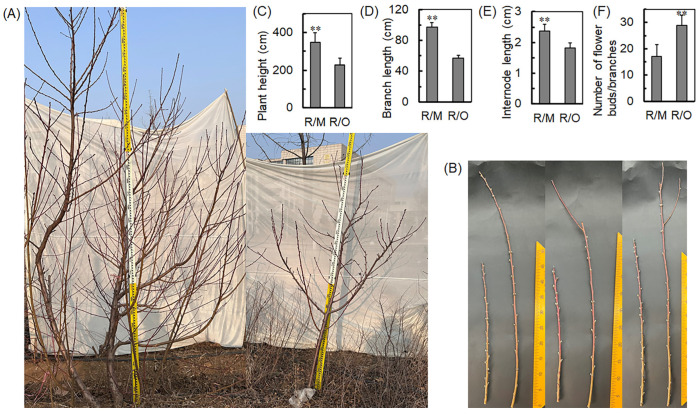
(**A**) Phenotypic comparison between R/M and R/O. (**B**) Branch phenotype of R/M and R/O. (**C**) Plant height, (**D**) branch length, (**E**) internode length, and (**F**) number of flower buds of R/M and R/O. The statistical significance of mean differences was evaluated using Student’s *t*-test, with significance levels denoted as ** *p* < 0.01.

Ruipan 4 was used as the scion and grafted onto *Amygdalus persica* and *Cerasus humilis*, respectively. The results indicate that rootstocks significantly influence the growth vigor of grafted peach trees (Figure 1). Compared to R/M (Ruipan 4/*Amygdalus persica*), R/O (Ruipan 4/*Cerasus humilis*) significantly reduces the growth height of peach trees, evidenced by notable decreases in plant height (Figure 1A,C), branch length (Figure 1B,D), and internode length (Figure 1B,E). Furthermore, compared to R/M, R/O increases the number of flower buds by 62.5% (Figure 1B,F).

### 2.2. Differentially Expressed Genes (DEGs) between R/O and R/M

**Figure 2:**
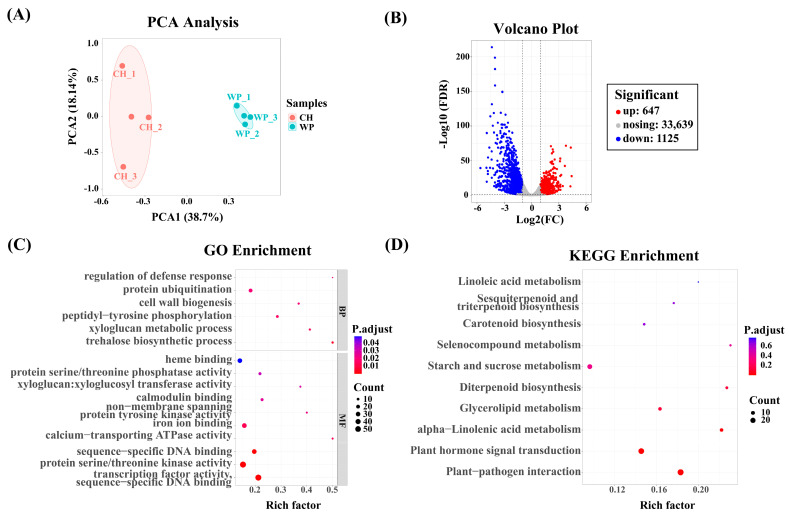
(**A**) PCA of the transcriptomes of R/O and R/M. (**B**) Statistical analysis of the number of DEGs between R/O and R/M. (**C**) GO enrichment analysis. The *p*-values are represented on the *x*-axis, while the GO terms are shown on the *y*-axis, and the size of each circle indicates the number of genes. The top 16 enriched GO terms are displayed in descending order of significance based on their *p*-values. The size of each circle represents the number of genes associated with the GO term, while the color of the circle reflects the *p*-values. (**D**) KEGG enrichment analysis. The *p*-values are represented on the *x*-axis, while the KEGG pathways are shown on the *y*-axis, and the size of each circle indicates the number of genes. The enriched KEGG terms are shown in the order of *p*-values.

To reveal the potential molecular networks involved in the dwarfing of peach trees mediated by *C. humilis* rootstocks, RNA-seq was conducted on leaves of R/O and R/M. Principal component analysis (PCA) demonstrates that the three biological replicates of R/M and R/O cluster together, indicating that the samples are reliable and consistent (Figure 2A). To understand the potential regulatory mechanisms, the number of DEGs was quantified, and genes with a |log_2_ FC| > 1 were selected for further analysis. The results indicate that there are 1772 differentially expressed genes between R/O and R/M, with 647 genes upregulated and 1125 genes downregulated (Figure 2B).

For further analysis, we identified 1772 DEGs for Gene Ontology (GO) and Kyoto Encyclopedia of Genes and Genomes (KEGG) analysis. The GO enrichment analysis revealed that the DEGs were significantly enriched in molecular functions, including transcription factor activity, sequence-specific DNA binding (GO:0003700), calcium-transporting ATPase activity (GO:0005388), and iron ion binding (GO:0005506) (Figure 2C). The ten most representative KEGG pathways are illustrated in Figure 2D, with the most significant being alpha-linoleic acid metabolism (ko00592), plant hormone signal transduction (ko04075), and plant–pathogen interactions (ko04626).

### 2.3. Weighted Gene Co-Expression Network Analysis (WGCNA Analysis) 

**Figure 3:**
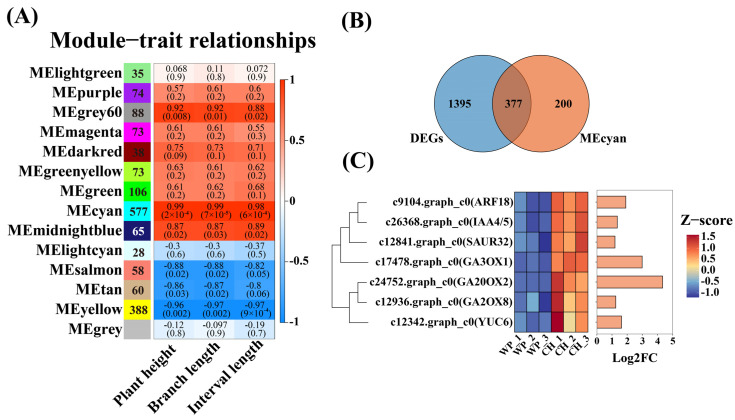
(**A**) WGCN heatmap showing correlations between traits and genes within modules. Each row represents a module, and each column corresponds to a specific trait. The correlation coefficients between modules and traits are denoted by the colors and text within the cells at the intersections (indicating *p*-values). Positive correlations are shown in red while negative correlations are shown in blue. (**B**) Venn diagram illustrating the overlap of 377 genes between the MEcyan module and DEGs. (**C**) Analysis of the expression patterns of 377 hormone-related differentially expressed genes in R/O and R/M.

To investigate the gene regulatory networks involved in the dwarfing process of peach trees mediated by *C. humilis* rootstocks, a comprehensive transcriptome-wide WGCNA was conducted on all expressed genes (15,540 genes), leading to the identification of key genes within the network. Co-expression modules were constructed using the Pearson correlation coefficient of gene expression in all samples, resulting in a total of 14 modules (Figure 3A). Each grid in Figure 3A is color-coded based on statistical significance, with each grid labeled with two numbers: the correlation coefficient and the *p*-value. The leftmost side of the grid represents each module and the number of genes enriched. The analysis revealed that there were 577 genes enriched in the MEcyan module, which is significantly correlated with plant height, branch length, and internode length, exhibiting a correlation coefficient of up to 0.99 (Figure 3A). As a result, the genes within the MEcyan module are deemed as a priority for future investigation. Further analysis revealed that 377 genes within the MEcyan module are differentially expressed (Figure 3B). Among these 377 genes associated with plant height, branch length, and internode length, studies focused on genes related to auxin (ARF18, IAA4/5, SAUR32, YUC6) and gibberellins (GA3ox1, GA2ox2, GA2ox7) (Figure 3C). Importantly, the transcription levels of GA2ox-related genes significantly increased. Since GA2ox plays a crucial role in the degradation of active GAs, the metabolism of GAs and the expression of GA2ox genes may be linked to the dwarfing mechanism of peach trees mediated by *C. humilis*.

### 2.4. PpGA2ox7 Regulates GA Levels in Peach Trees

**Figure 4:**
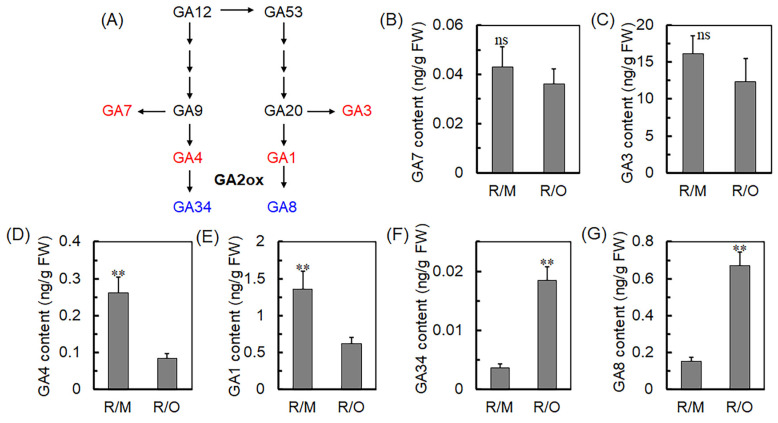
(**A**) GA biosynthesis pathway. The third stage of GA biosynthesis follows a distinct metabolic pathway, starting with GA12 or its isomer GA53, which then diverges into two separate pathways. GA12 and GA53 are oxidized to produce inactive intermediates GA9 and GA20, as well as biologically active GAs such as GA1, GA3, GA4, and GA7. Ultimately, GA1 and GA4 are converted into the inactive forms GA8 and GA34, respectively. Red represents bioactive GAs and blue represents inactive GAs. Content of (**B**) GA7, (**C**) GA3, (**D**) GA4, (**E**) GA1, (**F**) GA34, and (**G**) GA8 in R/O and R/M. The statistical significance of mean differences was evaluated using Student’s *t*-test, with significance levels denoted as ** *p* < 0.01 and ^ns (no significance)^ *p* > 0.05.

To further understand the role of GAs in the dwarfing mechanism of peach trees mediated by *C. humilis* rootstocks, the GA biosynthesis pathway was used as the starting point (Figure 4A). The levels of GA1, GA3, GA4, GA7, GA8, and GA34 in R/O and R/M were measured (Figure 4B–G). The results indicate that the levels of GA1, GA3, GA4, and GA7 in R/O are significantly lower than those in R/M (Figure 4B–E), while the levels of GA8 and GA34 are significantly higher relative to R/M (Figure 4F,G). These findings suggest that *C. humilis* rootstocks can alter the levels of six GAs in the biosynthesis pathway, potentially through the expression of the GA2ox family. It was observed that *PpGA2ox7* were significantly upregulated in *C. humilis* rootstocks (Figure 3C), which might influence the dwarfing effect of the rootstocks. Consequently, further investigations into *PpGA2ox7* were conducted in this study. 

### 2.5. PpGATA21 Directly Binds and Activates PpGA2ox7 Expression

**Figure 5:**
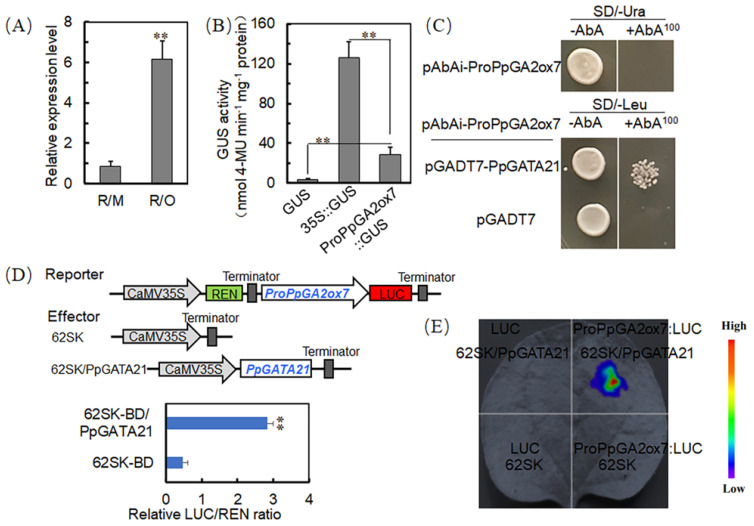
(**A**) Analysis of *PpGA2ox7* gene expression patterns in R/O and R/M. (**B**) GUS used as a negative control and 35S::GUS as a positive control to validate the activity of the *PpGA2ox7* promoter. (**C**) PpGATA21 protein directly binds to the PpGA2ox7 promoter, cloned into the pABAi vector. An empty pGADT7 vector is used as a negative control, and the interaction is validated through the growth of transformed yeast on SD/-Leu/AbA^100^ medium. (**D**) Schematic of PpGATA21 effector vector and LUC reporter vector (**top**), and results of the dual-luciferase assay analyzed using the LUC/REN ratio (**bottom**), data presented as mean (±SE). The statistical significance of mean differences was evaluated using Student’s *t*-test, with significance levels denoted as ** *p* < 0.01. (**E**) Representative dual-luciferase images, control LUC/REN ratio normalized to 1 (*n* = 6).

RT-qPCR results demonstrate that *PpGA2ox7* is significantly upregulated in R/O (Figure 5A), suggesting it may respond to the dwarfing effect mediated by *C. humilis* rootstocks. GUS assay results indicate that, compared to the negative control, the *PpGA2ox7* promoter activates the expression of the GUS reporter gene (Figure 5B), demonstrating that this promoter can initiate gene expression.

The *PpGA2ox7* promoter was used as bait; yeast one-hybrid (Y1H) screening identified 37 positive colonies, among which three encoded the gene LOC18792628. This gene showed high homology with the *Arabidopsis* GATA-type transcription factor GATA21, and was therefore named PpGATA21 (XM_020560109, LOC18792628).

To investigate whether PpGATA21 regulates *PpGA2ox7*, a recombinant plasmid was transformed into Y1H Gold yeast competent cells using a Y1H. Yeast strains co-transformed with the empty pGADT7 and pGADT7-PpGATA21/pABAi-PpGA2ox7 exhibited growth on the SD/-Leu medium, indicating successful transformation. Yeast strains transformed with the empty pGADT7 vector were unable to grow on SD/-Leu plates containing 100 ng/mL AbA, whereas strains transformed with pGADT7-PpGATA21/pABAi-PpGA2ox7 exhibited growth (Figure 5C), the results indicate that PpGATA21 can directly bind to the *PpGA2ox7* promoter.

The *PpGA2ox7* promoter was cloned into the pGreenII0800-LUC vector, resulting in the reporter plasmid PpGA2ox7-LUC0800. The full-length PpGATA21 was cloned into the 62SK vector, resulting in the effector plasmid PpGATA21-62SK (Figure 5D). The recombinant plasmid was created and then introduced into the *A. tumefaciens* GV3101 strain along with the pSoup helper plasmid. For the experimental group, the combination (ProPpGA2ox7/LUC + PpGATA21/62SK) was used, with dual empty vector controls (LUC + 62SK) and single empty vector controls (LUC + PpGATA21/62SK and ProPpGA2ox7/LUC + 62SK) serving as comparisons. The ratio of LUC to REN demonstrates that PpGATA21 positively regulates the expression of *PpGA2ox7* (Figure 5D,E).

### 2.6. PpTATA21 Is a GATA-Type Transcriptional Activator

**Figure 6:**
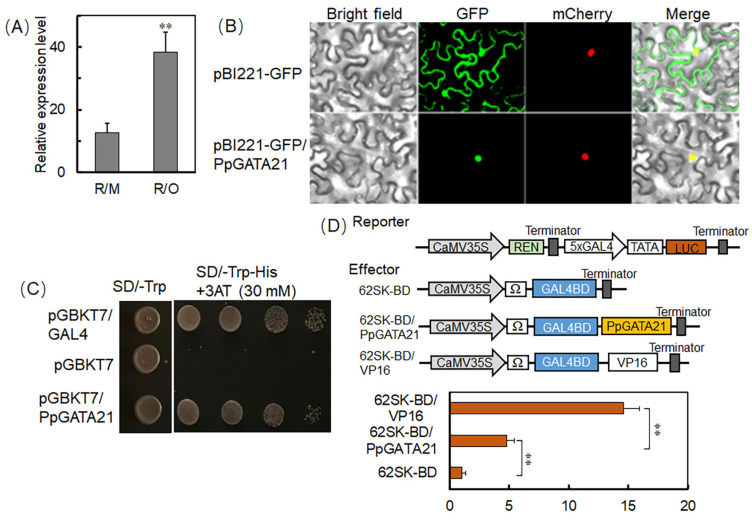
(**A**) Analysis of PpGATA21 gene expression patterns in R/O and R/M. (**B**) Subcellular localization of PpGATA21 in *N. benthamiana* leaf cells, using pBI221-GFP as the empty vector control. mCherry served as the nuclei localization marker. The signals of bright field, GFP and mCherry were detected. Merged images show colocalization of GFP and mCherry signals. (**C**) Transcriptional activation of PpGATA21 in yeast cells demonstrated by growth on SD/-Trp-His + 3AT medium; pGBKT7/GAL4 used as the positive control and pGBKT7 empty vector as the negative control. (**D**) Schematic of the PpGATA21 effector vector and LUC reporter vector (**top**), with dual-luciferase assay results showing the transcriptional activation activity of the PpGATA21 transcription factor (**bottom**). Analysis based on the LUC/REN ratio. Data presented as mean (±SE). The statistical significance of mean differences was evaluated using Student’s *t*-test, with significance levels denoted as ** *p* < 0.01.

RT-qPCR results demonstrate that, compared to R/M, the expression of PpGATA21 in R/O is significantly increased (Figure 6A), suggesting that PpTATA21 is involved in the dwarfing process of peach trees induced by *C. humilis*. Subcellular localization revealed that the fluorescence of PpTATA21 overlaps with the nuclear-localized mCherry, indicating that this gene is localized to the nucleus (Figure 6B). Further analysis was conducted to assess the transcriptional activity of PpTATA21, using pGBKT7 as the negative control and pGBKT7/GAL4 as the positive control. Yeast cells containing pGBKT7-PpGATA21 were able to grow on plates containing 3AT, indicating that PpGATA21 possesses transcriptional activation activity (Figure 6C). Further, the LUC/REN ratio of 62SK/BD-PpTATA21 was significantly higher than that of the negative control 62SK/BD, as determined by the dual-luciferase reporter (DLR) (Figure 6D).

## 3. Discussion

Plums are particularly focused on rootstock breeding as donors for ecological compatibility or resistance [25,26]. Reports suggest that among all *Prunus* species, *Prunus cerasifera* shows the highest diversity in both ecological adaptation and morphological traits [27]. Compared to other types of rootstocks, *C. humilis* is particularly favored for its dwarf stature and vigorous growth. Therefore, exploring and analyzing the dwarfing effect of *C. humilis* on scions is of significant importance [28]. The findings of this study contribute to a deeper understanding of the working mechanisms of *C. humilis* and how it mediates the dwarfing of peach scions. In this study, *C. humilis* significantly reduced the growth height of peach trees and can increase the number of flower buds by 62.4% (Figure 1). The increase in the number of flower buds on scions is attributed to the fact that scions on dwarf rootstocks can cease branch growth earlier and enhance the assimilation and allocation of nutrients toward bud production [3,29]. RNA-seq data identified a total of 1772 DEGs in C. humilis. GO and KEGG analysis highlighted the significance of transcription factor activity and plant hormone signal transduction in the process of *C. humilis*-mediated peach scion dwarfing (Figure 2). Furthermore, WGCNA revealed 577 genes enriched in the MEcyan module, which were closely associated with scion plant height, branch length, and internode length, with 377 genes showing differential expression (Figure 3). Previous research has demonstrated that weak citrus rootstocks TO and FD exhibit reduced plant height, plant weight, and internode length compared to the strong CC rootstock [30]. 

Increasing evidence suggests that plant hormones play a role in the dwarfing process of plants [31,32,33,34]. The key gene *DkGA2ox1* in persimmon dwarf disease acts as a signaling stimulus affecting the levels of GAs in scions, and overexpression of *DkGA2ox1* results in a typical dwarf phenotype [22]. An upregulation of GAox genes has been observed in dwarf lychee varieties, suggesting that GAs play a crucial role in mediating significant differences between dwarf and robust lychee varieties [35]. Consistently, our study found that *C. humilis* rootstocks alter the synthesis pathway of six types of GAs in peach scions, with significant reductions in the levels of GA1, GA3, GA4, and GA7. The GA2ox family genes are essential in the GA biosynthetic pathway, as illustrated in Figure 4A. The research pinpointed PpGA2ox7, a crucial gene in GA metabolism, as significantly linked to the alterations in plant height, branch length, and internode length influenced by *C. humilis* rootstock (Figure 3C). This suggests a potential role in inducing dwarfism in the scion through GA metabolism. Similarly, the transcription factor ABI4 directly binds to promoters and activates the transcription of GA2ox7, and mutations in GA2ox7 rescue the dwarf phenotype caused by reduced GA levels in plants overexpressing ABI4 [36]. These results indicate that *C. humilis* rootstocks induce dwarfing in peach scions by reducing the levels of GA, which is attributed to increased expression of *PpGA2ox7*.

Transcription factors are essential for the regulation of plant growth, development, stress responses, and hormone signaling [37,38,39]. The expression of hormone-related metabolic genes is continually proven to be regulated by transcription factors [40]. Specifically, the transcription factor JUB1 directly inhibits the biosynthesis genes GA3ox1 and DWARF4 (DWF4), leading to a decrease in levels of GAs and brassinosteroids (BRs). This reduction leads to the development of a characteristic phenotype associated with GA/BR deficiency, including shortened hypocotyls, dwarfism, delayed blossoming, and infertility in male plants [40]. Y1H screening was utilized in this study to identify the transcription factor PpGATA21 of PpGA2ox7. The research revealed that PpGATA21 is a nuclear-localized GATA-type transcription factor, and its expression is upregulated by R/O induction. PpGATA21 is primarily described as an inhibitor within the gibberellin signaling pathway [41]. Transcriptional activation analysis confirmed that PpGATA21 exhibits transcriptional activity (Figure 4). Previous studies have shown that in addition to GA signaling, GATA-type transcription factors also participate in other pathways, including those involving abscisic acid [42] and cytokinins [43]. This study utilized Y1H and DLR techniques to confirm that PpGATA21 plays a positive role in regulating the expression of PpGA2ox7, thereby influencing the involvement of GAs in the dwarfing mechanism of peach scions grafted onto *C. humilis* rootstocks (Figure 3). Similar to GATA21, the poplar transcription factor PdeGATA3 suppresses GA biosynthesis by inhibiting the expression of PdeGA20ox, leading to dwarfism in poplar trees [44].

**Figure 7:**
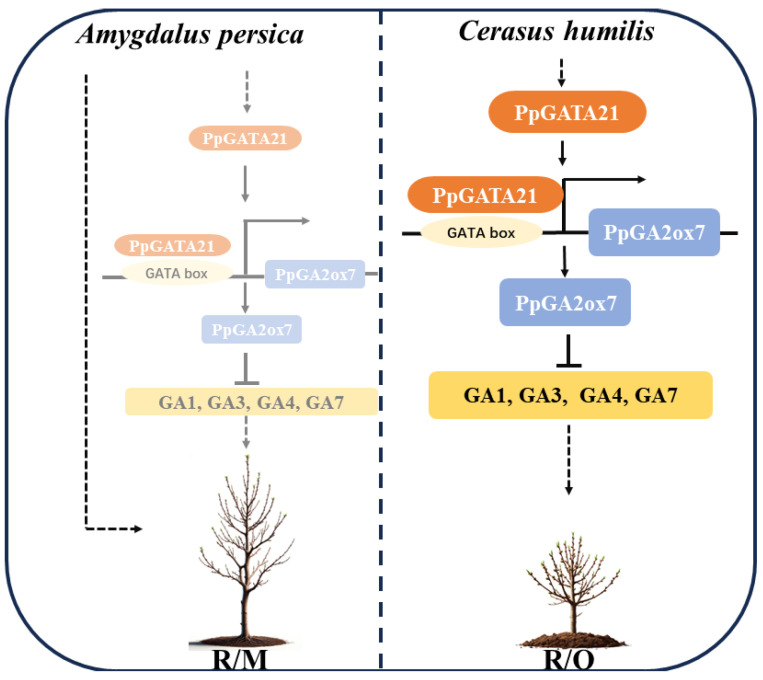
Functional model of the PpGATA21-*PpGA2ox7* regulatory module in the dwarfing of peach trees mediated by *C. humilis*. On the left, when grafted onto *A. persica* rootstocks, the GA content in peach trees does not change, leading to increased plant height as the dwarfing pathway mediated by the PpGATA21-PpGA2ox7 module is not activated. On the right, grafting onto *C. humilis* rootstocks induces the expression of the PpGATA21 gene. The PpGATA21 transcription factor positively regulates the expression of *PpGA2ox7* by binding to its promoter. The *PpGA2ox7* gene acts as a negative regulator of GA biosynthesis. Arrows indicate positive regulatory effects between components. Solid arrows indicate activation, whereas blunt-ended arrows indicate inhibition. The dashed arrow remains to be further experimentally confirmed.

Our working model, as shown in Figure 7, portrays the positive regulation of *PpGA2ox7* expression by PpGATA21 transcription under the influence of *C. humilis* rootstocks. On the other hand, *C. humilis* rootstocks suppress GA biosynthesis by activating the expression of *PpGA2ox7* (Figure 1 and Figure 7). Since optimal hormone levels are essential for regular plant growth, the regulation of *PpGATA21* by GAs could play a vital role in the accurate management of dwarfing.

## 4. Materials and Methods

### 4.1. Fruit Materials and Treatments

Ruipan 4 was used as the scion and grafted onto *Amygdalus persica* and *Cerasus humilis*, respectively. The graft combinations were R/O (Ruipan 4/*Cerasus humilis* (*Bge.*) *Sok*) and R/M (Ruipan 4/*Amygdalus persica* L.), sourced from Luoyang, Henan (34°37′ N, 112°38′ E). In the experimental region, the annual average temperature is 14.2 °C. Traditional field management practices are followed, including the application of organic fertilizer in the fall and urea before bud break in the spring. Additionally, flooding irrigation is utilized both before spring bud break and after autumn leaf fall, with adjustments based on weather conditions. In 2020, one-year-old *C. humilis* and *A. persica* rootstocks were grafted with Ruipan 4 scions. The grafted trees experienced growth during the period from 2020 to 2024. Sampling was performed on the current year’s shoots of young trees three years after grafting (May 2023) for RNA-seq, RNA extraction, and hormone content analysis. The study included sampling from four distinct trees and the experiment was conducted three times, totaling 24 trees (R/O and R/M). Each tree was situated 2.0–2.4 m apart.

### 4.2. Measurement of Plant Growth Parameters

Measurement data for scion growth were recorded, including plant height (cm), branch length (cm), average internode length (cm), and the number of flowers. (1) Plant height and branch length were measured using a tape measure; (2) The number of nodes on the branches above the grafted scion was calculated by counting; (3) Internode length was calculated by dividing the scion length by the total number of nodes; (4) The number of flowers on the scion was determined using the counting method.

### 4.3. RNA Extraction and RNA-Seq Analysis

The extraction of total RNA was carried out with the FastPure Cell/Tissue Total RNA Isolation Kit (Vazyme, Nanjing, China), followed by quantification of RNA concentration using a Nanodrop 2000 (Thermo Scientific, Waltham, MA, USA). Sequencing was performed by Beijing Biomarker Technologies Co., Ltd. (Beijing, China). Principal component analysis (PCA) was conducted on all expressed genes using count data. The results of PCA were visualized with the R packages factoextra (1.0.7) and FactoMineR (2.11). Differentially expressed genes (DEGs) were identified using DESeq2 (1.42.1), with a |log_2_ fold change (log_2_FC)| ≥ 1.0 set as the threshold for differential gene screening and an adjusted *p*-value (*p*adj) < 0.05, resulting in the identification of 1772 differential genes. Weighted Gene Co-expression Network Analysis (WGCNA) was performed using R software (version 4.0.2) to analyze all 15,540 expressed genes, requiring a TPM (Transcripts Per Million) > 1 and the soft threshold was set at 6. We utilized TBtools (2.070) for visualizing WGCNA and generating heat maps [45] and to illustrate the overlapping relationship between all DEGs (1772) and MEcyan in WGCNA. R software was utilized to conduct Gene Ontology (GO) enrichment analysis on 1772 differential genes, identifying significantly enriched GO pathways with corrected *p*-values < 0.05. A script written in R language was employed to perform Kyoto Encyclopedia of Genes and Genomes (KEGG) enrichment analysis on the same set of 1772 differential genes. Multiple testing correction was applied using the false discovery rate method. KEGG pathways with corrected *p*-values < 0.05 were significantly enriched. The expression of all genes in the transcriptome are provided in Supplementary Table S2.

### 4.4. RT-qPCR

RNA was reverse transcribed into cDNA using the NovoScript^®^ Plus All-in-one 1st Strand cDNA Synthesis SuperMix (gDNA Purge) (Novoprotein, Suzhou, China). RT-qPCR was conducted in accordance with the instructions provided by the Vazyme ChamQ Universal SYBR qPCR Master Mix (Vazyme, Nanjing, China) and carried out on a CFX96 Real-Time PCR Detection System 574 (Bio-Rad, Hercules, CA, USA). Data analysis was conducted using the 2^−ΔΔCt^ method, with *18S rRNA* [46] serving as the internal reference gene. All experiments included three biological replicates. The primers utilized for RT-qPCR are provided in Supplementary Table S1. The melt curve in RT-qPCR experiment is provided in Supplementary Figure S1.

### 4.5. Quantification of Endogenous Hormones

Plant hormone contents were measured according to methods described in previous studies [47]. Each sample was weighed to 0.2 g and ground into powder using liquid nitrogen. Hormone contents were then measured with a Qtrap 5500 System mass spectrometer (AB Sciex, Shanghai, China), using standards from Sigma Chemical Co. (St. Louis, MO, USA). Each experiment was conducted with three biological replicates.

### 4.6. Quantitative GUS Assay

The promoter of *PpGA2ox7* was inserted into the vector pC0390-35S-GUS (PpGA2ox7::GUS) for the purpose of activating the GUS reporter gene. The GUS empty vector was used as the negative control, and the 35S::GUS vector served as the positive control. *Nicotiana benthamiana* leaves were immersed in the inoculated solution and placed under vacuum until water-soaked lesions appeared. After treatment, the collected leaf samples were pulverized into powder using liquid nitrogen, and the measurement of GUS activity was conducted with the use of a β-Glucosidase (β-GC) Activity Assay Kit (Boxbio, Beijing, China) for the GUS gene.

### 4.7. Yeast One-Hybrid (Y1H)

A cDNA library was constructed from mRNA extracted from the leaves of peach trees. Yeast one-hybrid screening was conducted using the Matchmaker Gold Yeast One-Hybrid Library Screening System (Clontech, San Francisco, CA, USA). The full-length sequence of PpGATA21 was cloned into the pGADT7 vector (pGADT7-PpGATA21), and subsequently transformed into bait-reporter cells. An empty pGADT7 vector was used as the control. Screening was conducted on SD/-Leu and supplemented with 100 ng/mL aureobasidin A (SD/-Leu + AbA^100^). The *PpGA2ox7* promoter was inserted into the pAbAi vector (pAbAi-ProPpGA2ox7) and transformed into Y1H Gold cells to establish bait-reporter cells. Screening was performed on SD/-Ura medium and supplemented with 100 ng/mL aureobasidin A (SD/-Ura + AbA^100^).

### 4.8. Transcriptional Activation Analysis in Yeast Cells

The CDS sequence of PpGATA21 was inserted into the pGBKT7 plasmid (pGBKT7/PpGATA21), and the plasmid was transformed into Y2H Gold cells. The pGBKT7-GAL4 plasmid served as the positive control, and the empty pGBKT7 vector was used as the negative control. Transformed yeast cells were plated on SD/-Trp and SD/-Trp-His + 3AT (50 mM) selective media.

### 4.9. Subcellular Localization Analysis

The full-length coding sequence of PpGATA21 (excluding the stop codon) was amplified from leaf cDNA, cloned into the pBI221-GFP vector, and used to create a pBI221-GFP/PpGATA21 fusion construct. Subsequently, the fusion construct was co-transformed with the nuclear marker mCherry into *N. benthamiana* leaves, and fluorescence signals were then observed using a laser scanning confocal microscope (Olympus, Tokyo, Japan).

### 4.10. Dual-Luciferase Reporter (DLR)

The promoter sequence of *PpGA2ox7* was cloned into the pGreenII0800-LUC vector to generate a reporter construct. The CDS of PpGATA21 was cloned into the pGreen II 62-SK plasmid to produce an effector construct, with pGreenII 0800-LUC and pGreen II 62-SK serving as negative controls. Constructs containing reporter and effector genes were transformed into *Agrobacterium tumefaciens* strain GV3101 (pSoup, St. Louis, MO, USA), and subsequently injected into *N. benthamiana* leaves. The Dual-Luciferase Reporter Assay System (Promega, Madison, WI, USA) was used to measure dual-luciferase activity in the injected *N. benthamiana* leaves.

### 4.11. Statistical Analysis

Data were statistically analyzed using Microsoft Excel software (2019). Statistical significance between two samples was assessed using Student’s *t*-test (** *p* < 0.01).

## 5. Conclusions

The dwarfing effect of *C. humilis* on peach scions is pronounced, and it also increases the number of flower buds. This study delves into a less explored area, investigating the impact of grafting at the molecular level. The result indicates that *C. humilis* enhances the expression of the GA metabolic gene *PpGA2ox7* by promoting the expression of PpGATA21, thereby reducing the levels of GA and consequently leading to the dwarfing of peach scions (Figure 7). Through the modulation of GA levels, *C. humilis* rootstock shows promise as a dwarfing rootstock for peach trees, offering valuable insights for future researchers in selecting suitable rootstocks for breeding programs or establishing modern orchards.

## Data Availability

The original contributions presented in the study are included in the Supplementary Materials; further inquiries can be directed to the corresponding author.

## Supplementary Materials

The supporting information can be downloaded at: https://www.mdpi.com/article/10.3390/ijms25137402/s1.
