# Supplementary material for: PpGATA21 Enhances the Expression of PpGA2ox7 to Regulate the Mechanism of Cerasus humilis Rootstock-Mediated Dwarf in Peach Trees
Source: Int J Mol Sci. 2024 Jul 5;25(13):7402. doi: 10.3390/ijms25137402 (PMC11242874; doi:10.3390/ijms25137402)
Supplement: Supplementary file 1 [file ijms-25-07402-s001.zip › Supplementary Table S1. The sequences of the primers used in these experiments.docx]

Table S1. The sequences of the primers used in these experiments

| Primer name | oligonucleotide primers | Purpose or vector |
| --- | --- | --- |
| qPpGA2ox7-F  qPpGA2ox7-R | TGTTAAGCCAATTCCGAACACTCT  CTCCGAATCACACTGCCTTCAC | qRT-PCR |
| qPpGATA21-F  qPpGATA21-R | CATGTGGTGGACGCCATAAGC  ACCTTCCTCTTCTCCTCCTCCT | qRT-PCR |
| 18S rRNA-F  18S rRNA-R | GGTCAATCTTCTCGTTCCCTT  TCGCATTTCGCTACGTTCTT | qRT-PCR |
| ProPpGA2ox7-F  ProPpGA2ox7-R | CTTCGTGGGACCCGAGGATGAA  CATAAGGACTGCCTTGGATTGGAGA | PCR |
| ProPpGA2ox7-GUS-F  ProPpGA2ox7-GUS-R | tgggcccggcgcgccaagcttCTTCGTGGGACCCGAGGATGAA  ggtggactcctcttagaattcCATAAGGACTGCCTTGGATTGGAGA | 0390GUS |
| ProPpGA2ox7-ABAi-F  ProPpGA2ox7-ABAi-R | ttgaattcgagctcggtaccCTTCGTGGGACCCGAGGATGAA  tacagagcacatgcctcgagCATAAGGACTGCCTTGGATTGGAGA | pABAi |
| pGADT7-PpGATA21-F  pGADT7-PpGATA21-R | gccatggaggccagtgaattcATGATGACACCAGTGTATCTGAACCCA  cagctcgagctcgatggatccTCAAGAGTGATTATTGATTAGGCCAC | pGADT7 |
| ProPpGA2ox7-LUC-F  ProPpGA2ox7-LUC-R | gtcgacggtatcgataagcttCTTCGTGGGACCCGAGGATGAA  cgctctagaactagtggatccCATAAGGACTGCCTTGGATTGGAGA | pGreenII0800-LUC |
| pGreenII62-SK/PpGATA21-F  pGreenII62-SK/PpGATA21-R | caggaattcgatatcATGATGACACCAGTGTATCTGAACCCA  gtcgacggtatcgatTCAAGAGTGATTATTGATTAGGCCAC | pGreenII62-SK |
| PpGATA21-GFP-F  PpGATA21-GFP-R | agagaacacgggggactctagaccATGATGACACCAGTGTATCTGAACCCA  cttctcccttacccatggtaccAGAGTGATTATTGATTAGGCCAC | pBI221-GFP |
