# Supplementary figures and images for: PpGATA21 Enhances the Expression of PpGA2ox7 to Regulate the Mechanism of Cerasus humilis Rootstock-Mediated Dwarf in Peach Trees
Source: Int J Mol Sci. 2024 Jul 5;25(13):7402. doi: 10.3390/ijms25137402 (PMC11242874; doi:10.3390/ijms25137402)

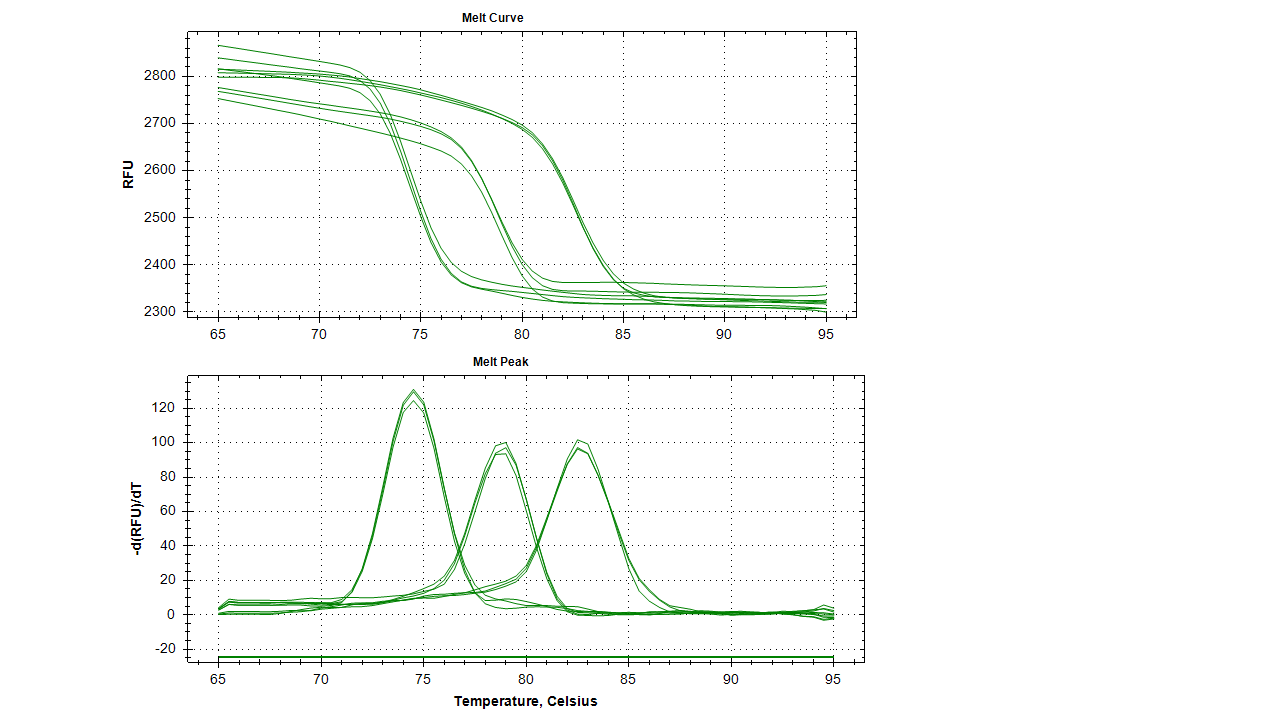

Supplement: Supplementary file 1 [file ijms-25-07402-s001.zip › Supplementary Figure S1. Melt curve in RT-qPCR experiment.png]
